# Myasthenia gravis with dysphagia as the first symptom and misdiagnosed as anxiety-depressive state: a case report

**DOI:** 10.3389/fimmu.2026.1751111

**Published:** 2026-07-10

**Authors:** Kariman Aili, Mayinuer Maimaiti

**Affiliations:** 1 Neuroimmunology Department, Neurology Diagnosis and Treatment Center, People’s Hospital of Xinjiang Uygur Autonomous Region, Urumqi, China; 2 Xinjiang Clinical Research Center for Stroke and Neurological Rare Disease, Urumqi, China

**Keywords:** anxiety-depressive disorder, dysphagia, FcRn antagonist, misdiagnosis, myasthenia gravis

## Abstract

**Background:**

Myasthenia gravis (MG) is a rare autoimmune disorder that affects the neuromuscular junction, with a global prevalence of approximately 30 cases per 100,000 individuals. Although dysphagia occurs as an initial symptom in 6%–15% of patients, isolated dysphagia as the sole presenting and predominant manifestation is exceedingly rare, posing a significant diagnostic challenge. Furthermore, a complex bidirectional relationship exists between dysphagia and psychiatric conditions such as anxiety and depression, which often leads to misdiagnosis. Approximately 20% of patients with MG are refractory to conventional immunosuppressive therapy. The novel FcRn antagonist efgartigimod rapidly and sustainably reduces pathogenic IgG levels, providing a new therapeutic option for refractory generalized MG.

**Case presentation:**

A 33-year-old previously healthy woman presented with fluctuating dysphagia and a sensation of a pharyngeal foreign body, exhibiting diurnal variation characterized by improvement in the morning and worsening in the evening. Symptom onset coincided with a family quarrel and subsequently intensified after an upper respiratory tract infection. Multiple laryngoscopies, gastrointestinal endoscopies, and brain magnetic resonance imaging (MRI) revealed no abnormalities. Initially misdiagnosed with an anxiety-depressive disorder, she experienced only partial and transient improvement with citalopram. Two subsequent acute exacerbations triggered by infections led to a complete inability to swallow. Neurological examination indicated dysarthria, choking when drinking, and mild distal limb weakness, assessed at 4+/5 on the Medical Research Council scale. The neostigmine test yielded a strong positive result, and serum anti-acetylcholine receptor (AChR) antibodies were detected at a titer of 1:10, while repetitive nerve stimulation (RNS) remained normal. Chest computed tomography (CT) revealed no thymoma. The patient was diagnosed with moderate generalized MG characterized by AChR-antibody positivity and classified as MGFA class IIIb. Following the failure of initial immunosuppressive therapy, which included prednisone at 30 mg daily, pyridostigmine at 60 mg three times daily, and azathioprine at 25 mg daily, the patient underwent two cycles (totaling 8 infusions) of the FcRn antagonist efgartigimod. Marked improvement in dysphagia was observed, as evidenced by a decrease in the MG-Activities of Daily Living (MG-ADL) score from 8 to 2 and a reduction in the Quantitative Myasthenia Gravis (QMG) score from 10 to 1. At the 6-month follow-up, the patient achieved complete clinical remission, with both MG-ADL and QMG scores reaching 0, and attained minimal manifestation status, while prednisone was tapered to 5 mg daily.

**Conclusion:**

This case indicates that isolated fluctuating dysphagia warrants consideration of MG, even in the presence of psychosocial stressors or partial response to psychiatric medications. A negative RNS does not exclude MG, especially in bulbar-onset cases. Clinicians should prioritize organic etiologies and perform neostigmine and anti-AChR testing to avoid delayed diagnosis. Furthermore, efgartigimod is effective for refractory bulbar MG, enabling rapid remission and corticosteroid tapering.

## 1 Introduction

MG is an autoimmune disorder affecting the neuromuscular junction, with a global prevalence of approximately 30 per 100,000 individuals (1), and an incidence of about 0.68 per 100,000 in China (2). The pathophysiology primarily involves specific autoantibodies targeting antigens at the neuromuscular junction, resulting in fluctuating muscle weakness and fatigability (3). Although MG is typically regarded as sporadic, epidemiological data indicate a notable familial aggregation; approximately 5–7% of MG patients report a positive family history, significantly exceeding the expected prevalence in the general population (4). The principal clinical manifestations of MG include binocular diplopia, ptosis, and generalized symptoms due to the involvement of limb, ocular, bulbar, and respiratory muscles (5).

Dysphagia and dysarthria are relatively common in MG, with Walander first documenting this connection in 1959 (6). Subsequent research has indicated that 6%–15% of patients may initially present with dysphagia (7). However, isolated dysphagia as the sole initial and predominant symptom is exceedingly rare and often goes unreported. These atypical symptoms lack specificity, leading to significant diagnostic challenges. Studies have revealed that individuals with bulbar−onset MG experience a lower quality of life compared to those with non−bulbar onset (*p=0.01)* (8), and are at a higher risk of severe complications such as compromised nutritional status, increased risk of aspiration, and airway blockage, ultimately contributing to elevated mortality and morbidity rates (9). Hence, prompt identification of MG at symptom onset is crucial for enhancing patient outcomes.

A complex relationship exists between dysphagia and psychiatric conditions, including anxiety and depression (10). Both depression and MG are more prevalent in women and are associated with immune dysregulation. Acetylcholine (ACh) signaling plays a role in the pathogenesis of both MG and depression; ACh levels are elevated in patients with depression, and blockade of acetylcholinesterase (AChE) can induce depressive symptoms (11). In some individuals, dysphagia may trigger or exacerbate depression, while depression may, in turn, worsen dysphagia (12). Furthermore, anxiety and depression double the mortality risk associated with MG (13).

The diagnosis of MG typically relies on a combination of characteristic clinical features, including fluctuating weakness, a positive neostigmine test, the presence of serum anti-AChR, and RNS findings. Approximately 80% of patients with MG exhibit detectable serum anti-AChR antibodies (14). Treatment options encompass pharmacotherapy, immunotherapy, plasmapheresis, and thymectomy. While conventional immunosuppressive agents, such as glucocorticoids, azathioprine, tacrolimus, and mycophenolate mofetil, effectively manage symptoms in most patients, around 20% remain unresponsive or intolerant, categorizing them as having refractory MG (15). Efgartigimod, a novel FcRn antagonist, inhibits FcRn-mediated IgG recycling and enhances the clearance of pathogenic IgG. This agent rapidly alleviates clinical symptoms in patients with generalized MG, significantly reduces serum IgG levels, and supports long-term disease control when used in conjunction with immunosuppressants (16).

## 2 Case presentation

**Figure 1:**
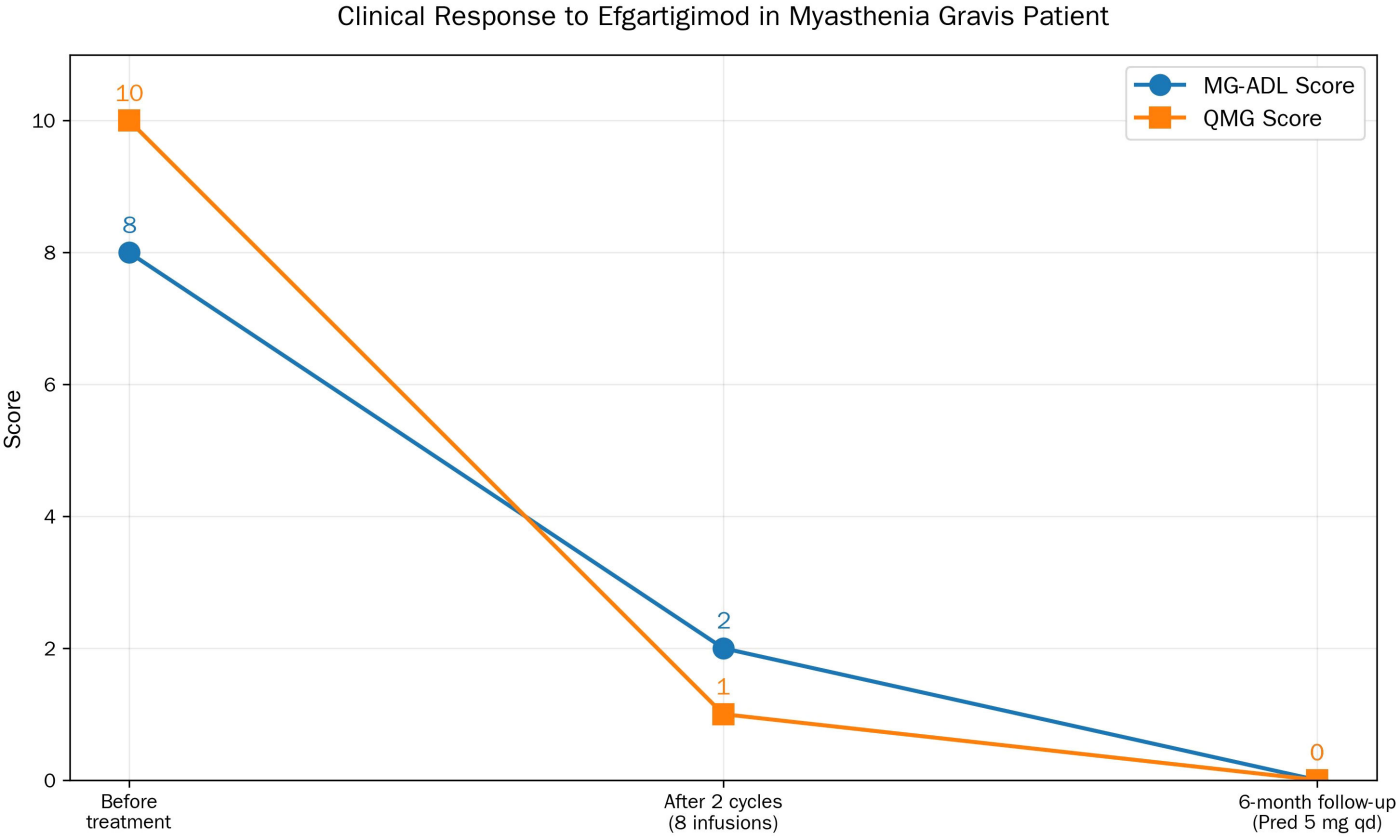
Clinical scale scores before and after treatment.

**Figure 2:**
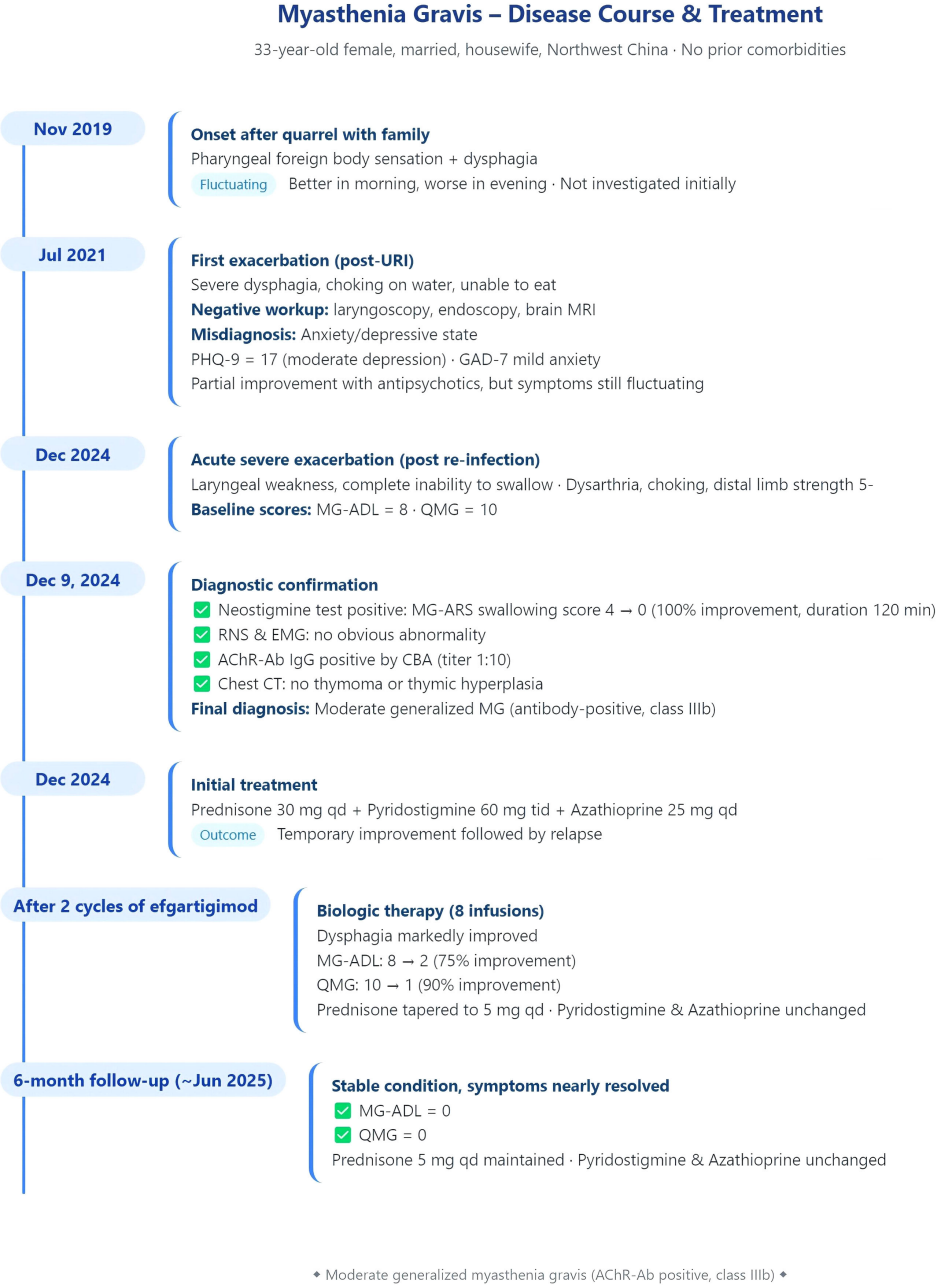
Timeline of disease course.

A 33-year-old married housewife of Han ethnicity from Northwest China, with no prior comorbidities, presented in November 2019 with a sensation of a foreign body in the pharynx and dysphagia following a family quarrel. The symptoms were fluctuating and diurnal, improving in the morning but worsening in the evening, and were initially disregarded. In July 2021, the symptoms intensified after an upper respiratory tract infection, presenting as severe dysphagia, choking while drinking, and an inability to eat, accompanied by persistent fluctuations. Multiple laryngoscopies, gastrointestinal endoscopies, and a brain MRI conducted at an outside hospital revealed no significant abnormalities. The patient was subsequently referred to psychiatry and diagnosed with an anxiety-depressive state. During the admission evaluation, the Patient Health Questionnaire-9 (PHQ-9) score (17) indicated moderate depression, while the Generalized Anxiety Disorder-7 (GAD-7) score (18) indicated mild anxiety. She was prescribed citalopram at a dosage of 40 mg once daily, which resulted in only partial and transient improvement in her dysphagia, with symptoms continuing to fluctuate persistently. In December 2024, the patient’s dysphagia acutely worsened following an infection, manifesting as laryngeal weakness and a complete inability to swallow. Neurological examination revealed dysarthria, choking during drinking, and distal limb weakness (4+/5 Medical Research Council grade), with no additional abnormalities noted. The neostigmine test yielded a strongly positive result, with the MG-ARS swallowing score improving from 4 to 0 (a 100% improvement sustained for 120 minutes). Electromyography and RNS showed no abnormalities. A cell-based assay (CBA) detected a positive anti-AChR at a titer of 1:10 (**Table 1**). Chest CT scans showed no thymoma or thymic hyperplasia. The differential diagnoses encompassed anxiety-depressive disorder, globus pharyngeus, motor neuron disease, and structural lesions of the pharynx or esophagus. All potential diagnoses were ruled out through negative results from endoscopy, imaging, and electrophysiological assessments. The final diagnosis was moderate generalized MG (AChR-antibody positive, MGFA class IIIb). Prior to treatment, the Myasthenia Gravis Activities of Daily Living Scale (MG-ADL)score (19) was 8, and the Quantitative Myasthenia Gravis (QMG) score (20) was 10. Initial treatment consisted of prednisone at 30 mg daily, pyridostigmine at 60 mg three times daily, and azathioprine at 25 mg daily; the azathioprine dose was increased to 50 mg daily after 2 weeks. Symptoms initially improved but subsequently relapsed. conventional immunosuppressive therapy failed due to symptom fluctuation and relapse. The FcRn antagonist efgartigimod was then administered for two cycles (totaling 8 infusions). Dysphagia showed significant improvement, with the MG-ADL score decreasing to 2 (a 75% improvement) and the QMG score decreasing to 1 (a 90% improvement). At the 6-month follow-up, the patient remained stable, with near-complete resolution of symptoms. Prednisone was tapered to 5 mg daily, while the doses of pyridostigmine and azathioprine remained unchanged. Both MG-ADL and QMG scores were 0 (**Figure 1**), indicating achievement of MGFA minimal manifestation status (MMS). The patient experienced considerable distress due to long-term dysphagia and recurrent misdiagnoses. Following treatment with efgartigimod, she regained the ability to swallow independently, resumed her daily activities, and reported substantial satisfaction with the clinical outcomes (**Figure 2**).

**Table 1:** Serum autoantibody profile.

Antibody	Result	Reference	Method
Anti-AChR IgG	1:10 (positive)	Negative	CBA
Anti-MuSK IgG	Negative	Negative	CBA
Anti-LRP4 IgG	Negative	Negative	CBA
Anti-RYR1 IgG	Negative	Negative	CBA
Anti-Titin IgG	Negative	Negative	ELISA

## 3 Discussion

Approximately 6%–15% of patients with MG present with dysphagia as an initial symptom; however, isolated dysphagia as the sole presenting and predominant feature is exceedingly rare (7). Patients with MG experience impaired neuromuscular transmission, and severe stressors—such as serious infections, surgical procedures, or psychological trauma—can disrupt normal neurotransmission (21), thereby upsetting the delicate balance and exacerbating MG (22). Psychiatric comorbidities are prevalent among individuals with MG, with anxiety and depression being the most common (23). The MG-ADL and QMG scores are significantly correlated with anxiety and depression in MG patients (*p<0.05)*. Studies indicate that, after adjusting for confounding variables, each 1-point increase in the MG−ADL score elevates the risk of anxiety and depression by 22.5% and 18.5%, respectively; similarly, each 1-point increase in the QMG score raises the risk of anxiety by 11.9% and depression by 15.7% (24). Sneddon et al. reported that approximately 30% of MG patients are initially misdiagnosed with a psychiatric disorder, often necessitating a prolonged period to achieve an accurate diagnosis, as symptoms can be easily mistaken for those of depression, hypochondriasis, or conversion disorder, resulting in significant delays in diagnosis and treatment (25).

In this case, the patient experienced dysphagia following severe familial conflict and subsequently underwent evaluation at a psychiatric clinic. The classic symptoms of MG, including fluctuating weakness (diurnal variation), fatigability, and dysphagia, closely resemble the somatic manifestations of mood disorders, such as persistent fatigue and depression (26). Notably, the patient’s dysphagia improved after the administration of citalopram. However, the effects of anxiolytics and antidepressants on MG remain inconsistent; benzodiazepines are generally considered relatively contraindicated or should be used cautiously in MG due to their skeletal muscle relaxant properties (27). Certain selective serotonin reuptake inhibitors (SSRIs), including fluoxetine and citalopram, can be safely employed to treat anxiety and depression in patients with MG without exacerbating weakness (28). The subjective improvement in dysphagia observed in this patient following citalopram treatment was likely attributable to reduced somatic amplification resulting from mood relief, rather than a direct reversal of MG pathophysiology. These factors complicate the relationship between anxiety-depressive states and dysphagia, thereby increasing the diagnostic challenges associated with MG.

The negative result of RNS in this patient presented a significant diagnostic challenge. The patient primarily exhibited dysphagia, affecting areas innervated by cranial nerves IX–XI, while conventional RNS testing sites, such as the deltoid and trapezius, demonstrate low sensitivity for medullary lesions. This limitation often results in false-negative outcomes, which accurately reflect the atypical phenotypic characteristics of MG in this instance. Benatar’s systematic review of 20 international studies established that RNS possesses a specificity of 95% and a sensitivity of approximately 80% in generalized MG (29). Despite the elevated false-negative rate associated with electrophysiological testing, the diagnosis of generalized MG remains robustly supported by clinical manifestations, a positive neostigmine test, serological confirmation, and treatment response.

The objectives of MG treatment include alleviating symptoms, decreasing antibody production, and preventing crises. Although the patient’s symptoms improved with a regimen of oral prednisone at 30 mg daily combined with azathioprine at 50 mg daily, fluctuations in symptoms persisted. According to international consensus guidelines, the treatment goal for MG is to achieve MMS or better, defined as being asymptomatic or exhibiting no functional impairment, with only mild weakness observed during manual muscle testing, while adverse events should not exceed grade 1 (30). The neonatal FcRn is crucial for prolonging the serum half-life of immunoglobulin G (IgG). Inhibition of FcRn disrupts IgG binding, accelerates its catabolism, and lowers pathogenic autoantibody levels, thus providing a therapeutic benefit (31). Research indicates that FcRn antagonists are safe, well-tolerated, and associated with improved survival in MG patients. These antagonists rapidly enhance clinical symptoms in individuals with generalized MG, significantly reduce serum IgG levels, and promote long-term disease control when used in conjunction with immunosuppressants (16). In this case, two cycles of efgartigimod followed by six months of maintenance therapy resulted in MG-ADL and QMG scores of 0, along with a tapering of prednisone to 5 mg daily, strongly supporting efgartigimod as an optimized therapeutic option for MG.

This patient endured a diagnostic delay of five years, resulting in significant physical and psychological harm. The extended misdiagnosis stemmed from both clinical factors and fluctuating symptoms, which contributed to inconsistent follow-up. Additionally, attribution bias related to psychological triggers, vague symptom descriptions, and limited local medical resources further complicated the situation. To mitigate misdiagnosis and reduce patient suffering, clinicians should prioritize the exclusion of organic diseases in patients presenting with dysphagia as either the primary or predominant symptom. Furthermore, they should incorporate multimodal immunotherapy alongside psychological interventions, while also offering clinical warnings and strategies tailored for primary care settings.

To elucidate the novelty of this case, we conducted a systematic review of the literature concerning MG misdiagnosed as psychiatric disorders, identifying eight directly relevant articles, including case reports, case series, and retrospective studies. Nicholson et al. (32) were the first to systematically address the issue of “psychiatric” misdiagnosis in MG, highlighting that patients who presented with fatigue and limb weakness were frequently misdiagnosed with conversion disorder or depression. Gasparini et al. (33) described a patient with bulbar-onset MG, in whom comorbid anxiety resulted in a four-year diagnostic delay and whose condition deteriorated following benzodiazepine treatment. Oguz-Akarsu et al. (34) reported that approximately 12% of patients with atypical MG presentations were initially misdiagnosed with a psychiatric disorder, with dysphagia and dysarthria identified as common misleading features. Falso et al. (35) documented an overall misdiagnosis rate of approximately 18% for MG, with anxiety and depression being among the most frequently reported incorrect diagnoses. Moreover, Gavrilov et al. (23) and Kulaksizoglu (26) have examined the high comorbidity rate of depression and anxiety in MG, which can reach 30–50%. They highlighted that these mood disorders may obscure or imitate typical fluctuating weakness, leading to diagnostic delays. While rare, cases presenting with isolated dysphagia as the primary symptom and being misdiagnosed as anxiety or depression are noteworthy. Only two closely comparable cases have been reported: one by Gasparini et al. (33), which had additional symptoms and a positive RNS; and a recent case report by Lusvarghi et al. (36) describing isolated dysphagia masking MG without FcRn antagonist therapy involvement.

This case illustrates several uncommon and noteworthy clinical features that have seldom been reported in combination: a diagnostic delay of 5 years, persistently negative RNS findings that underscore the low sensitivity of conventional RNS for bulbar-onset MG, and a successful response to efgartigimod following the failure of conventional immunosuppressive therapy. To our knowledge, the coexistence of isolated fluctuating dysphagia, persistently negative RNS, a prolonged misdiagnosis as an anxiety-depressive disorder, and a favorable response to efgartigimod has not been well documented in the literature. The strengths of this report include a 5-year long-term follow-up, a clear diagnostic workflow, detailed objective scoring (MG-ADL, QMG), and real-world evidence of efgartigimod in refractory bulbar MG with negative RNS.

## 4 Limitations

This study is constrained by its classification as a single case report, which limits the ability to draw definitive conclusions regarding the general efficacy of efgartigimod in patients with atypical bulbar-onset myasthenia gravis. The findings presented herein are exploratory and serve to generate hypotheses rather than provide conclusive evidence. Further large-scale, prospective controlled studies are necessary to validate the effectiveness, safety, and long-term outcomes of FcRn antagonists in refractory MG patients who exhibit isolated dysphagia and normal electrophysiological test results. Additionally, the long-term prognosis of this patient beyond the 6-month follow-up period remains to be determined.

## 5 Conclusion

This case details a patient with AChR-antibody-positive generalized MG who initially presented with isolated dysphagia. The patient experienced a misdiagnosis of anxiety-depressive disorder for five years, had persistently false-negative results on RNS, and demonstrated a positive response to efgartigimod following the failure of conventional treatments. Such a combination of findings is rarely reported in the literature. This case underscores three key points: (1) isolated fluctuating dysphagia should prompt consideration of MG, even in the presence of psychosocial stressors; (2) a negative RNS does not rule out MG, particularly in cases with bulbar onset; and (3) efgartigimod may prove effective for refractory generalized MG with bulbar involvement.

## Data Availability

The raw data supporting the conclusions of this article will be made available by the authors, without undue reservation.
